# Comparability of hemoglobin A1c level measured in capillary versus venous blood sample applying two point-of-care instruments

**DOI:** 10.1186/s40200-014-0094-1

**Published:** 2014-10-30

**Authors:** Tahereh Keramati, Farideh Razi, Ali Tootee, Bagher Larijani

**Affiliations:** Endocrinology and Metabolism Research Center, Endocrinology and Metabolism Clinical Sciences Institute, Tehran University of Medical Sciences, Tehran, Iran; Diabetes Research Center, Endocrinology and Metabolism Clinical Sciences Institute, Tehran University of Medical Sciences, Tehran, Iran

## Abstract

**Background:**

The present study is designed to evaluate the validity of the measurement of capillary blood hemoglobin A1c levels in comparison with venous blood hemoglobin A1c.

**Methods:**

The data of this cross-sectional study are collected from a sample of 45 Iranian diabetic patients referred to one particular laboratory for the assessment of HbA1c level during a period from April to December 2013. Venous and simultaneous capillary blood samples were obtained from each subject for measurement of hemoglobin A1c levels. Both samples were tested using two different NGSP certified systems: CERA STAT 2000 (Ceragem Medisys Inc) and NycoCard Reader II (Axis-Shield).

**Results:**

The mean hemoglobin A1c in venous and capillary blood samples measured using CERA STAT 2000 assays were 6.30 ± 1.68% and 6.34 ± 1.65% respectively (p = 0.590). However, when NycoCard Reader II assay was employed, the mean hemoglobin A1c in venous and capillary blood samples were 6.73 ± 1.35% and 6.92 ± 1.50% (p = 0.007). Moreover, a strong correlation was observed between venous and capillary hemoglobin A1c levels with Pearson’s concordance correlation coefficients of 0.96 and 0.94 with the use of NycoCard Reader II and CERA STAT 2000 assays respectively. Application of CERA STAT 2000 demonstrated to be of a considerably higher value using the ROC curve analysis assay (AUC = 0.991). Also, similar analysis by using NycoCard Reader II assay demonstrated that capillary hemoglobin A1c measurement had high value for differentiation of uncontrolled from controlled blood glucose level (AUC = 0.935).

**Conclusion:**

It was demonstrated that capillary hemoglobin A1c measurement had a considerably high value for differentiating between poorly-controlled and well-controlled blood glucose levels.

## Introduction

Currently, millions of people are living with diabetes mellitus and suffer from its adverse and even life-threatening consequences all around the world and a 58% increase in its prevalence by 2025 is predicted [1,2]. According to a recent American Diabetes Association, the cost involved in the management of diabetic patients is $245 billion in 2012, which is remarkably higher in comparison with non-diabetic individuals [3].

Iran is one of the main focuses of diabetes in the Middle East and the overall prevalence of diabetes mellitus and impaired fasting glucose among Iranian population has estimated to be 7.7% and 16.8% respectively [4]. This figure appears to be significantly lower in comparison with the reported prevalence in neighboring countries such as Oman with the prevalence rate of 16.1% [5].

Venous blood sampling is currently the gold standard for the assessment of blood glucose levels. However, this method of sampling is hindered by several factors such as cumbersome transportation of the samples to laboratories and noncompliance of patients with venous blood sampling [6,7]. In contrast, capillary blood glucose testing, using portable point of care devices, is hailed as an alternative method to venous blood sampling considering its better compliance, lower cost, and its potential for self-monitoring [8]. Nonetheless, there are some doubts about its diagnostic accuracy in comparison with venous blood sampling [9].

In this regard, many studies are designed and carried out to compare the validity of testing capillary blood glucose in comparison with venous blood glucose. However, to the best of our knowledge, to date, there has been no study for comparison of venous and capillary blood hemoglobin A1c levels with the same assay.

Hence, the present study is designed to assess the comparability between capillary blood hemoglobin A1c and venous blood hemoglobin A1c in diabetes management.

## Methods

### Study population

The data of this cross-sectional study were collected from a group of Iranian patients who were referred to one particular laboratory to assess blood glucose control during a period from April to December 2013. In this regard, 45 patients agreed to participate in the study. The median age of the participants was 47 years, ranging from 18 to 66 years. There were 24 males and 21 females.

### Study measurement

Health checks including anthropometric measurements as well as both venous and capillary blood sampling were performed at the survey sites by specifically trained nurses. Venous blood samples were obtained from the subjects for the assessment of venous hemoglobin A1c blood levels, and a capillary blood sample was simultaneously obtained from a fingertip for the assessment of hemoglobin A1c concentration. The venous blood samples were collected and stored in vacuum tubes containing K2EDTA (BD company). The two mentioned assays, NycoCard Reader II assay (Axis-Shield Co.) and CERA STAT 2000 (Ceragem Medisys Inc), were employed for measurement of hemoglobin A1c levels in both venous and capillary samples which were measured in duplicate. The levels of hemoglobin A1c was measured using the Boronate affinity method in both assays.

It needs to be mentioned that the study was approved by the Ethics Committee of Endocrinology metabolism Research Institute and all participants gave written informed consent.

### Statistical analysis

All analyses were performed according to the guideline of Clinical and Laboratory Standards Institute (CLSI EP9-A2). The results of the paired venous and capillary blood hemoglobin A1c measurements were used for statistical analysis. Mean (standard error, SE) and 95% confidence intervals, as well as the percentages, are presented as necessary. The Pearson correlation coefficient (r) was determined by linear regression method. The accuracy of capillary blood sampling was compared with venous blood capillary at the cutoff point 6.5% of hemoglobin A1c using the ROC curve. For the statistical analysis, the statistical software SPSS version 20.0 for windows (SPSS Inc., Chicago, IL) was used.

## Results

**Figure 1:**
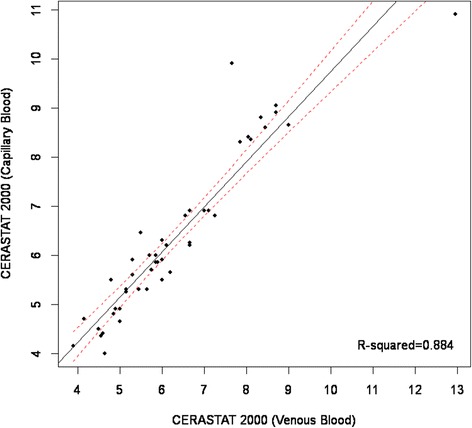
**Regression analysis of capillary and venous blood; Cerastat 2000.**

**Figure 2:**
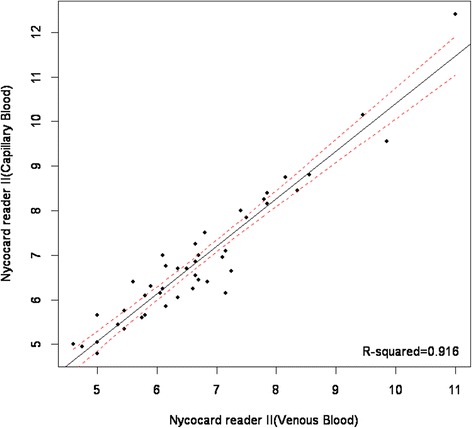
**Regression analysis of capillary and venous blood; Nycocard reader II.**

The mean hemoglobin A1c levels in venous and capillary blood samples measured using CERA STAT 2000 assay were 6.30 ± 1.68% (ranged 3.90 to 12.95%) and 6.34 ± 1.65% (ranged 4.00 to 10.90%) respectively with no significant differences demonstrated by t-test (p = 0.590). However, when NycoCard Reader II assay was employed, the mean hemoglobin A1c in venous and capillary blood samples were 6.73 ± 1.35% (ranged 4.60 to 11.00%) and 6.92 ± 1.50% (ranged 4.80 to 12.40%) respectively which was significantly lower in the former (p = 0.007). The Passing & Bablok Regression Equation for the relationship between venous hemoglobin A1c and capillary hemoglobin A1c was *Y = 0.92x + 0.56* using CERA STAT 2000 assay (Figure 1) and *Y = 1.07x-0.26* using NycoCard Reader II assay (Figure 2). A strong correlation was observed between venous and capillary hemoglobin A1c levels, with a Pearson’s concordance correlation coefficient of 0.96 (p = 0.007) and 0.94 (p = 0.59) when NycoCard Reader II assay and CERA STAT 2000 assay were used respectively. Table 1 shows a 100% concordance between capillary and venous hemoglobin A1c when CERA STAT 2000 assay was applied and a 81.0% concordance between capillary and venous hemoglobin A1c when NycoCard Reader II assay was used. ROC curve analysis demonstrated that when CERA STAT 2000 assay was used, capillary hemoglobin A1c measurement was of a considerably high value for differentiation between poorly-controlled and well-controlled diabetes (AUC = 0.991, 95% CI: 0.972 – 1.000, P < 0.001). Also, a similar analysis using NycoCard Reader II assay, demonstrated that capillary hemoglobin A1c measurement had also a high value for differentiation between poorly-controlled and well-controlled blood glucose levels (AUC = 0.935, 95% CI: 0.869 – 1.000, P < 0.001).

**Table 1:** **Agreement for the classification of patients using the same cut-off values for venous plasma laboratory and capillary blood samples**

	**Venous blood**			
**Capillary blood**	**HbA1c < 6.5%**	**HbA1c 6.5-8.0%**	**HbA1c > 8.0%**	**Total**
**NycoCard II assay**				
**HbA1c < 6.5%**	17 (81.0)	5 (27.8)	0	22 (48.9)
**HbA1c 6.5-8.0%**	4 (19.0)	9 (50.0)	0	13 (28.9)
**HbA1c > 8.0%**	0	4 (22.2)	6 (100)	10 (22.2)
**Total**	21 (100)	18 (100)	6 (100)	45 (100)
**Cerastat 2000 assay**				
**HbA1c < 6.5%**	28 (100)	2 (22.2)	0	30 (66.7)
**HbA1c 6.5-8.0%**	0	5 (55.6)	0	5 (11.1)
**HbA1c > 8.0%**	0	2 (22.2)	8 (100)	10 (22.2)
**Total**	28 (100)	9 (100)	8 (100)	45 (100)

## Discussion

Although various studies have investigated the difference between the mean capillary blood glucose levels and mean venous blood glucose levels, as mentioned in the introduction, our study can be considered as the first one to assess such a difference in regards to the measurement of mean venous and capillary blood hemoglobin A1c levels with same method.

The findings of our study demonstrated that when NycoCard Reader II assay was used there was a strong correlation between venous and capillary hemoglobin A1c levels, a finding less significant when another assay (CERA STAT 2000) was employed (correlation coefficient: 0.96 versus 0.94). Regardless of the association between hemoglobin A1c levels assessed in venous and capillary blood samples, capillary sampling demonstrated to be of high diagnostic value in both assays in terms of differentiation between poorly-controlled and well-controlled diabetes. Therefore, based on our findings, it can be suggested that due to the high concordance between venous and capillary Hb A1c levels as well as high discriminatory value, the use of capillary blood can be recommended to be applied in diabetes clinical settings for the assessment of diabetes control.

In some studies indicating significant difference in venous and capillary values, some documents were presented to explain this difference such as concentration of proteins and blood cells. In this regard, although some studies have suggested that there are negligible differences when a free flow of blood has been obtained, [10] others have shown definite differences in composition between skin puncture and venous blood samples in neonates, [11] children [12] and adults [13]. On the composition of capillary and venous blood sample, Kupke et al. [14] showed that total protein, bilirubin, calcium, sodium and chloride concentrations were significantly lower in capillary than in venous sample. There was also a tendency for glucose concentrations to be higher in capillary than in venous sample. With regard to difference in lipid and lipoprotein concentrations of capillary and venous blood samples, Kupke et al. [15] also showed that the concentrations of lipids and lipoproteins measured in capillary blood taken from young adults were significantly lower than in venous blood that may reflect differences in the morphological and hemodynamic conditions existing either in large veins or in the peripheral circulatory system. Also, some studies focused difference between venous and fingerstick capillary blood glucose values. Rasaiah et al. showed a typically quoted difference value up to 80 mg/dL between venous and fingerstick capillary blood glucose values one hour after ingestion of 100 grams of glucose [16].

Although some physicians prefer to apply capillary sampling method for assessing both serum glucose concentration and also hemoglobin A1c level due to its more facility and higher patients’ satisfaction, but some authors have already rejected the practice of the latter method and have recommended that venous sample be used for all glucose and A1c determinations [17]. In a recent editorial, glucose measurement in whole blood was considered anachronistic [18], however according to our survey, following the capillary sampling method can be considered as a suitable option especially in those conditions manifested by patients’ dissatisfaction to give venous sample for assessment of glucose control status.

In conclusion, we could show strong correlation between the value of hemoglobin A1c measured by applying capillary and venous blood samples by using NycoCard Reader II and CERA STAT 2000 assays. Also, in this study, high value of capillary blood sampling was revealed to discriminate uncontrolled from controlled blood glucose level and thus this type of sampling can be easily and correctly applied for measuring level of hemoglobin A1c.
